# Impact of learning curve on outcomes of cytoreductive surgery and hyperthermic intraperitoneal chemotherapy in colorectal cancer patients at a specialized center

**DOI:** 10.1590/1806-9282.20250905

**Published:** 2026-03-30

**Authors:** Vural Argın, Ömer Özduman, Ahmet Orhan Sunar, Gülten Çiçek Okuyan, Mustafa Duman, Erdal Polat

**Affiliations:** 1 University of Health Sciences, Kartal Koşuyolu Yüksek Ihtisas Training and Research Hospital – Istanbul, Türkiye.; 2 Haydarpasa Numune Training and Research Hospital – Istanbul, Türkiye.; 3 University of Health Sciences, Kartal Koşuyolu Yüksek Ihtisas Training and Research Hospital, Gastrointestinal Surgery Clinic – Istanbul, Türkiye.

**Keywords:** Cytoreductive surgery, HIPEC, Learning curve, Peritoneal carcinomatosis

## Abstract

**BACKGROUND:**

Cytoreductive surgery and hyperthermic intraperitoneal chemotherapy are promising treatments for colorectal cancer-related peritoneal metastases. The learning curve may influence surgical outcomes.

**OBJECTIVE:**

The aim of the study was to compare outcomes of cytoreductive surgery and hyperthermic intraperitoneal chemotherapy during the learning curve versus after surgical proficiency at a gastrointestinal cancer center.

**METHODS:**

A retrospective study of 83 patients treated between 2018 and 2023. Group 1 (n=42) included initial cases; Group 2 (n=41) represented the experienced phase. Operation time, organ resections, morbidity, transfusion needs, and survival were compared.

**RESULTS:**

Severe morbidity (Clavien-Dindo grade 3–4) was similar in both groups (G1: 21.4%, G2: 29.2%; p=0.947). No 30- or 90-day mortality occurred. Transfusions of ≥3 units were more frequent in G1 (p=0.003). Mean operative time was longer in G1 (8.5 vs. 7.2 h). More patients in G2 had multiple organ resections (p<0.001).

**CONCLUSION:**

The learning curve significantly influences perioperative outcomes in cytoreductive surgery and hyperthermic intraperitoneal chemotherapy procedures. As surgical experience increases, intraoperative transfusion requirements and operative time decrease, while the extent of resection improves. However, these improvements do not appear to translate into a survival benefit.

## INTRODUCTION

Cytoreductive surgery (CRS) and hyperthermic intraperitoneal chemotherapy (HIPEC) offer favorable outcomes in peritoneal metastases from colorectal cancer^1^. While chemotherapy alone yields a median survival of 16.3 months, CRS and HIPEC can extend it up to 40 months^2^. In specialized centers, 16% of patients achieving complete cytoreduction remain recurrence-free at 5 years^3^. First described by Sugarbaker in the early 1990s, CRS aims for macroscopic tumor clearance from peritoneal surfaces^4^. Complete resection (CC-0 or CC-1) often requires extensive procedures, leading to complication rates of 14–70% and perioperative mortality up to 8%, even in high-volume centers^5,6^. This high risk is largely linked to the learning curve; mortality decreases after 100–140 cases^7^. The early learning phase carries the highest risk, making surgical experience, protocols, and guideline adherence critical^8^.

Despite increasing use, data on CRS and HIPEC implementation and learning processes remain limited^9^. Many studies lack detail on team experience. In this study, we analyzed colorectal cancer patients with peritoneal metastases treated by a surgical team with over 10 years of exclusive gastrointestinal cancer experience. Patients were grouped by experience phase, and surgical and oncological outcomes were compared to assess the impact of the learning curve.

## METHODS

The study was approved by the University of Health Sciences, Kartal Koşuyolu Yüksek İhtisas Training and Research Hospital Ethics Committee (Approval No: 2024/18/998). We retrospectively analyzed 135 consecutive patients aged 18–75 who underwent curative-intent (CC-0 or CC-1) CRS and HIPEC for colorectal cancer-related peritoneal metastases between 2018 and 2023. Forty-five patients who received prophylactic HIPEC for T4 tumors and seven who were lost to follow-up were excluded. The remaining 83 patients were grouped chronologically: the first 42 as Group 1 (G1) and the next 41 as Group 2 (G2). Data were prospectively collected.

The CRS and HIPEC program began in 2018 with two senior surgeons and a multidisciplinary team. Standardized protocols were established based on Peritoneal Surface Oncology Group International (PSOGI) 2008 guidelines^10^. Staging included thoracoabdominal computed tomography (CT), with magnetic resonance imaging (MRI) for suspected liver metastases or mucinous tumors, and positron emission tomography–computed tomography (PET-CT) when needed. Tumor markers were evaluated. Diagnostic laparoscopy was used for uncertain resectability. Palliative procedures were performed for patients with Eastern Cooperative Oncology Group (ECOG) ≥2 or non-resectable metastases.

Patients were followed post-discharge by oncology and surgery teams. Recurrences were discussed by a tumor board. We compared perioperative outcomes, including hospital/intensive care unit (ICU) stay, operative time, surgical radicality, morbidity, and mortality, between learning-phase and post-learning-phase surgeries.

### Surgical technique

All patients received prophylaxis for deep vein thrombosis with low-molecular-weight heparin and anti-embolic stockings. Surgery began in the Lloyd-Davis position with a midline incision from xiphoid to symphysis pubis. Prior surgical scars were excised due to potential tumor implants. The Peritoneal Cancer Index (PCI) was used intraoperatively to assess disease burden. Disease-specific peritonectomy was performed, including procedures such as omentectomy, parietal/pelvic peritonectomy, bowel resections, hysterosalpingo-oophorectomy, splenectomy, antrectomy, and Glisson capsule resection, depending on disease extent. Optimal CRS was defined as a CC score of 0 or 1.

HIPEC followed CRS using a closed technique and perfusion machine. Chemotherapeutics included intraperitoneal oxaliplatin+intravenous 5-fluorouracil /leucovorin or intraperitoneal mitomycin C. After intra-abdominal temperature reached 42°C, chemotherapy was circulated for 90 min at 42–43°C via five drains, with a flow rate of 1,000 cc/min. Anastomoses were performed after HIPEC; in patients not requiring anastomosis, abdominal closure was completed beforehand.

### Statistical analysis

As this was a retrospective study including all eligible patients treated during the study period, no prior sample size calculation or power analysis was performed. Data were analyzed using Statistical Package for the Social Sciences (SPSS) v26.0. Categorical variables were expressed as frequencies (%), and continuous variables as mean±standard deviation, median, or min–max. Categorical comparisons used chi-square, and Fisher’s exact test was applied when any expected cell count was less than 5. Normality was assessed with the Shapiro-Wilk test. Non-normal data were analyzed using Mann-Whitney U. Survival was evaluated via Kaplan-Meier and log-rank tests. p<0.05 was considered significant.

## RESULTS

Eighty-three patients underwent CRS and HIPEC: 42 in G1 (learning period) and 41 in G2 (experienced period). Mean age was similar (G1: 53.9±10.8, G2: 52.8±12.9). G1 included 27 (64.3%) males and 15 (35.7%) females; G2 had 15 (36.6%) males and 26 (63.4%) females (p=0.012). Pathological diagnoses were similarly distributed between groups (p=0.494).

Clavien-Dindo grade 3–4 morbidity occurred in 9 (21.4%) G1 and 12 (29.2%) G2 patients (p=0.947). No grade 5 mortality was observed within 30 or 90 days. Intraoperative complications occurred in 2 patients per group (p=0.980); diaphragm injuries were seen in G1, while G2 had one diaphragm and one major vessel injury (Table 1). PCI≤11 was noted in 24 (57%) G1 and 29 (70%) G2; PCI ≥12 in 18 (43%) G1 and 12 (30%) G2 (p=0.36). CC-0 cytoreduction was achieved in 40 (95.2%) G1 and 38 (92.7%) G2; CC-1 in 2 (4.8%) G1 and 3 (7.3%) G2 (p=0.625) (Table 1). <3 units of erythrocyte transfusion were required in 17 (41.4%) G1 and 5 (11.9%) G2; ≥3 units in 4 (9%) G1 and 3 (7%) G2 (p=0.003). Mean operative time was longer in G1 (8.5±3.06 h vs. 7.2±2.71 h). G2 had significantly more cases with 1–3 organ resections (p<0.001). The mean hospital stay was 14.7±12.8 days (G1) vs. 12.6±10.7 days (G2) (p=0.197). ICU stay was shorter in G2 (1.43±0.92 vs. 2.07±3.08 days, p=0.022) (Table 1).

**Table 1:** Baseline demographic, histopathological, and operative parameters of patients undergoing cytoreductive surgery and hyperthermic intraperitoneal chemotherapy in Group 1 (learning period) and Group 2 (experienced period) patients.

Variables		Learning period (n=42) (n%)	Experienced period (n=41) (n%)	p-value
Gender	Male	27 (64.3)	15 (36.6)	**0.012**
	Female	15 (35.7)	26 (63.4)	
Age at surgery: years (range)		54 (24–73)	55 (25–71)	0.993
Primary tumor pathology	Adenocarcinoma well differentiated	6	9	0.494
	Adenocarcinoma moderately differentiated	8	9	
	Adenocarcinoma poorly differentiated	6	1	
	Mucinous adenocarcinoma	10	12	
	Signet ring cell carcinoma	1	1	
	Serous carcinoma	1	-	
	Neuroendocrine tumor	1	-	
	Others	9	9	
CC score	0	40 (95.2)	38 (92.7)	0.625
	1	2 (4.8)	3 (7.3)	
Clavien-Dindo grade 3 and 4 morbidities		9	12	0.947
Clavien-Dindo grade 5 mortality		0	0	
PCI score	≤11	24 (57%)	29 (70%)	0.36
	≥12	18 (43%)	12 (30%)	
Median surgery duration, hours (range)		8 (3.5–16)	7 (3–14)	**0.04**
Blood transfusion	<3 units	17 (41.4%)	5 (11.9%)	**0.003**
	≥3 units	4 (9%)	3 (7%)	
Median ICU stay, days (range)		1 (1–21)	1 (1–5)	0.071
Median duration of hospital stay, days (range)		12 (5–77)	9 (5–60)	0.197
No. of organs resected	0	12 (28.6%)	2 (4.9%)	**<0.001**
	1–3	27 (66.7%)	35 (92.6%)	
	4–5	3 (7.2%)	1 (2.4%)	

ICU, intensive care unit; CC, completeness of cytoreduction; PCI: Peritoneal Cancer Index. Bold values indicate statistically significant results (p<0.05).

**Figure 1:**
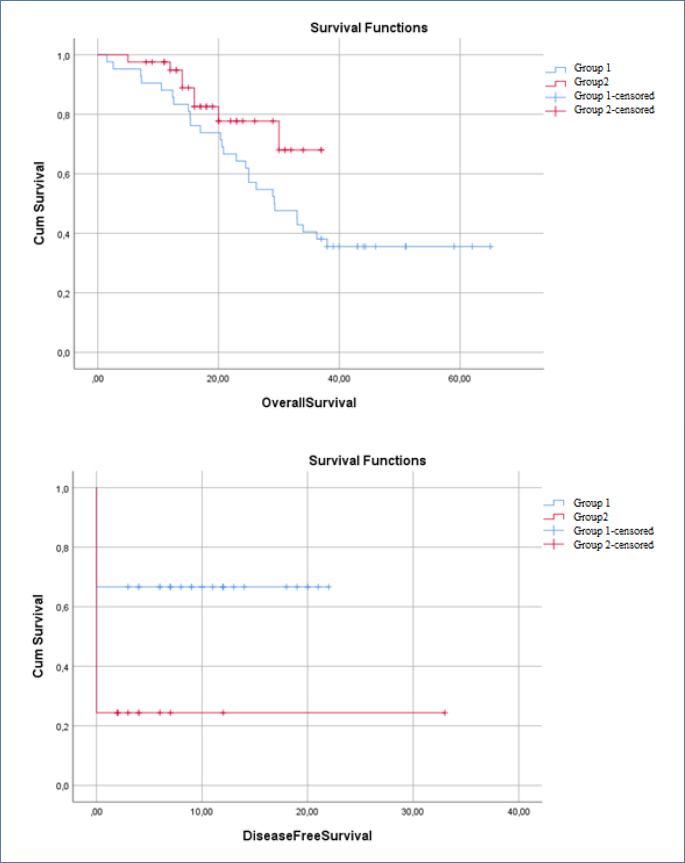
Overall survival, disease-free survival comparison between Group 1 (learning period) and Group 2 (experienced period) patients who underwent cytoreductive surgery and hyperthermic intraperitoneal chemotherapy.

Median overall survival was 29 months in G1 and 18 months in G2 (p=0.09) (Table 2). Disease-free survival was significantly longer in G1 (14.6 vs. 8 months, p<0.001) (Figure 1).

**Table 2:** Mean overall survival and disease-free survival times with minimum–maximum ranges in Group 1 and Group 2 patients.

	Mean±SD (Mo)	Minimum (Mo)	Maximum (Mo)	p-value
Overall survival				
Group 1	36.5±3.4	29.7	43.4	0.09
Group 2	31.3±1.7	27.9	34.7	
Disease-free survival				
Group 1	14.6±1.6	3.7	12.3	**<0.001**
Group 2	8±2.2	11.5	18.6	

p<0.05, Kaplan-Meier log rank, Mo: month, Group 1: learning period, Group 2: experienced period. SD: standard deviation. Bold values indicate statistically significant results (p<0.05).

## DISCUSSION

This study evaluated CRS and HIPEC outcomes based on surgical experience, comparing the learning and experienced periods. Surgical experience significantly affected operative time, extent of resection, and transfusion need, while morbidity, mortality, and survival outcomes were similar between groups.

Clavien-Dindo grade 3–4 morbidity rates and 30-/90-day mortality were comparable (p=0.947), suggesting that multidisciplinary support, standardized protocols (PSOGI), and surgical focus maintained safety even during the learning period^10^. Similar findings were reported by Saikia et al., where higher morbidity in the learning phase was not statistically significant^11^. Some studies even suggest lower morbidity during the early phase^12^.

PCI scores did not differ significantly between groups (p=0.36), with most patients having PCI≤11. This likely reflects consistent patient selection. Compared to literature, our scores were lower, possibly due to exclusive inclusion of colorectal cancer patients, while other studies include mesothelioma or ovarian cancers^13,14^. Complete cytoreduction (CC-0) was high in both groups (95.2 vs. 92.7%, p=0.625), which literature suggests requires experience over 40–180 cases^15^. Our team’s decade-long gastrointestinal cancer surgery experience and strict selection likely contributed to these rates.

Operative time was longer in G1 (p=0.04), reflecting caution during early procedures. Similar trends are reported in the literature, where increased experience shortens surgical time through better coordination and workflow^16,17^.

Organ resection was more extensive in G2 (p<0.001), indicating increased surgical confidence, skill, and intraoperative decision-making. Early in the learning phase, surgeons tend to be conservative. Literature supports that experienced teams can perform more aggressive resections, though impacts on outcomes must be weighed carefully^18,19^.

Blood transfusions were more frequent in G1 (p=0.003), likely due to cautious intraoperative management. As experience grows, teams adopt a more restrictive transfusion strategy^16^.

Hospital stay was similar between groups (p=0.197), while ICU stay was shorter in G2 (p=0.022), aligning partially with literature. Polanco et al. found no difference, while Chidambarasamy et al. reported shorter ICU stays in experienced periods^16,20^. Our results suggest that standardized postoperative care ensures consistency regardless of experience.

Although overall survival did not differ significantly (p=0.09), disease-free survival was longer in G1 (p<0.001). This may reflect patient selection differences or variations in systemic therapy. Literature shows mixed results; some studies link better survival to experience, while others find no correlation^21,22^.

## CONCLUSION

As experience with CRS and HIPEC has increased, both operative time and the need for transfusions have decreased, even though more extensive resections have been undertaken. Despite these improvements, overall survival has remained unchanged. The noted variation in disease-free survival emphasizes the importance of further studies aimed at optimizing surgical techniques and refining patient selection criteria, ideally within larger cohorts and with long-term follow-up.

## Data Availability

The datasets generated and/or analyzed during the current study are available from the corresponding author upon reasonable request.
